# A Label-Free Electrochemical Impedance Cytosensor Based on Specific Peptide-Fused Phage Selected from Landscape Phage Library

**DOI:** 10.1038/srep22199

**Published:** 2016-02-24

**Authors:** Lei Han, Pei Liu, Valery A. Petrenko, Aihua Liu

**Affiliations:** 1 Institute for Biosensing & In-Vitro Diagnostics, and College of Medicine, Qingdao University, 38 Dengzhou Road, Qingdao 266021, China; 2 Laboratory for Biosensing, Qingdao Institute of Bioenergy & Bioprocess Technology, Chinese Academy of Sciences, 189 Songling Road, Qingdao, 266101, China; 3 Department of Pathobiology, College of Veterinary Medicine, Auburn University, 269 Greene Hall, Auburn, Alabama 36849-5519, United States

## Abstract

One of the major challenges in the design of biosensors for cancer diagnosis is to introduce a low-cost and selective probe that can recognize cancer cells. In this paper, we combined the phage display technology and electrochemical impedance spectroscopy (EIS) to develop a label-free cytosensor for the detection of cancer cells, without complicated purification of recognition elements. Fabrication steps of the cytosensing interface were monitored by EIS. Due to the high specificity of the displayed octapeptides and avidity effect of their multicopy display on the phage scaffold, good biocompatibility of recombinant phage, the fibrous nanostructure of phage, and the inherent merits of EIS technology, the proposed cytosensor demonstrated a wide linear range (2.0 × 10^2^ − 2.0 × 10^8^ cells mL^−1^), a low limit of detection (79 cells mL^−1^, S/N = 3), high specificity, good inter-and intra-assay reproducibility and satisfactory storage stability. This novel cytosensor designing strategy will open a new prospect for rapid and label-free electrochemical platform for tumor diagnosis.

With the increasing cancer incidence and stubbornly high cancer mortality around the world, the early diagnosis of cancer and point-of-care monitor of cancer therapy have drawn great attention^1^,^2^. To date, various analytical methods including chemiluminescence^3^, quartz crystal microbalance biosensor^4^, surface plasmon resonance^5^, radiological examination^6^, endoscope^7^, computed tomography^8^, and electrochemical approaches has attracted growing interests for detection of cancer cells. Recently, electrochemical methods such as scanning electrochemical microscopy^9^, electrochemiluminescence^10^, electrochemical impedance spectroscopy (EIS)^11^, and electric cell-substrate impedance sensing^12^ have been developed. Among them, EIS has attracted great interests due to its remarkable advantages. First, EIS is an effective tool to sensitively and rapidly monitor the minute changes of the electrode interface. Second, unlike large amplitude perturbation techniques (such as cyclic voltammetry and differential pulse voltammetry), EIS is a nondestructive technique due to the small amplitude perturbation^13^. Last but most importantly, it is not needed to introduce a label during impedance measurement^14^. Therefore, by collecting EIS data of electrode interface at direct current^15^, the label-free electrochemical impedance cytosensors have been increasingly explored for clinical diagnosis of tumor, due to the inherent advantages such as label-free procedure, simple operation, high sensitivity, short analysis time, miniaturization, and potential of automation^16^.

Based on affinity interaction, label-free impedance cytosensor usually needs a kind of recognition element to selectively capture targets or analytes^14^, such as antibody^17^,^18^, lectin^19^,^20^, receptor protein^21^, DNA aptamer^22^, and carbohydrate^13^. For example, Maalouf *et al*. achieved label-free detection of *E. coli* by EIS using biotinylated polyclonal antibodies immobilized on the gold electrode (AuE)^18^. Hu *et al*. developed EIS cytosensor for quantitative determination of human BXPC-3 pancreatic cancer cells by monoclonal anti-carcinoembryonic antigen antibody as recognition element^23^. Li *et al*. explored indium tin oxide electrode covered with a monoclonal antibody for EIS sensing of *E. coli* without enzymatic signal amplification^17^. Cao *et al*. reported a cytosensor based on protein-inorganic hybrid nanoflowers conjugated with a targeting lectin molecule (*Sambucus nigra* agglutinin) for sensing human DLD-1 colon cancer cells^24^. Wan *et al*. fabricated an impedimetric cytosensor for label-free and rapid detection of *Desulforibrio caledoiensis* by immobilizing lectin (Concanavalin A, Con A) onto the surface of AuE^20^. Gamella *et al*. fabricated label-free screen-printed AuE for EIS detection of bacteria by the selective interaction of lectin (Con A) with carbohydrate component from *E. coli* cell surface^25^. The Labib’ group developed two EIS biosensors to separately detect *Salmonella enteritidis*^26^ and *Salmonella typhimurium*^22^ by highly specific DNA aptamer immobilized on Au nanoparticles/screen-printed carbon electrode. Guo *et al*. developed a label-free EIS biosensor for *E. coli* ORN 178 by self-assembling thiol-terminated α-mannoside and a thiol-terminated oligoethylene glycol “spacer” molecule on AuE^13^. However, some inherent disadvantages of the conventional recognition elements restrict their analytical applications. On one hand, protein recognition elements (antibody, lectin and receptor protein) and DNA aptamer, are complicated in preparation and purification, and lack the stability under a variety of monitoring conditions or long-term storage^27^. On the other hand, although carbohydrate exhibits the merits of good stability, it still involves complicated functionalization process and use of toxic organic reagents^28^. In addition, carbohydrate lacks specificity in comparison with other recognition elements. Therefore, it is highly desirable to explore facile, stable and specific molecular probes to overcome the limitations inherent in these typical recognition elements.

Phage display is a technology that allows expressing of polypeptide or protein on the surface of phage by inserting the corresponding foreign DNA into the genes encoding the coat proteins^29^. Since one of our co-workers, Petrenko constructed the first type f8/8 phage library, named “landscape phage library”, displaying billions of random octapeptides at the N-end of the major coat protein pVIII of fd-tet phage^30^, landscape phage libraries have become an effective source to obtain specific peptide-fused phages binding various analytes by biopanning (a biological evolutionary selection process)^31^. By displaying specific ligands, phage display technology has been widely applied on various fields, such as biomineralization^32^,^33^, epitope screening^34^, cancer diagnosis and therapy^35^,^36^, targeted drug delivery^37^, and biosensing^31^,^38^. The selected phages exhibit many merits such as multivalency, high specificity, excellent stability in extreme environments, and simple large-scale preparation without complicated purification procedures^39^. Additionally, the filamentous phages can be used as a “carrier” of specific polypeptides which reduce the challenge of polypeptides immobilization^40^,^41^,^42^,^43^.

To study the phage-based cytosensor, the SW620 colorectal carcinoma cells were used as the model targets, which were derived from metastatic lesions of colon cancer^44^. In our previous work, we obtained the phage fused with octapeptide (DDAGNRQP) by screening the f8/8 landscape phage library against SW620 human colorectal carcinoma cells. The bio-mimetic nanostructure was self-assembled from Au@Ag heterogeneous nanorods and specific fusion pVIII proteins, which were capable of bioimaging and photothermal therapy of targeted tumor^45^. In this work, a label-free EIS cytosensor for cancer cells was developed by immobilizing the above-mentioned specific octapeptide-fused phages on the electrode surface. The as-fabricated cytosensor was sensitive, selective, reliable and stable, which was benefitted from the inherent merits of phage-displayed specific octapeptides and the superiority of EIS. To the best of our knowledge, this is the first example of impedance cytosensor based on specific peptide-fused phages. This work will provide a new avenue to fabricate the phage-based sensors for detection of cancer cells.

## Results

### Spectrophotometric Estimation of SW620 Cell-Specific Phage Particles

The total number of SW620 cell-specific phages from the f8/8 phage library was determined spectrophotometrically. The purified phage solution was diluted with phosphate-buffered saline (PBS) for the required concentration. The typical UV spectrum of purified phage preparation is shown in Figure S1(Supporting Information). Based on the formula^41^: virions (vir)/ml = (A_269_ × 6 × 10^16^)/number of nucleotides in the phage genome, where A_269_ is the absorbance at 269 nm. For the recombinant phages used in this work (9198 nucleotides), absorbance unit (A_269_) = 6.52 × 10^12^ vir/ml was used to determine the concentration of phage particles in the solution (physical titer). The concentration of the as-prepared phage particles was calculated to be 9.13 × 10^13^ vir/ml.

### Fabrication of the Cytosensor

**Figure 1:**
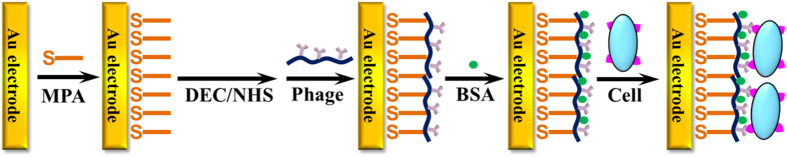
Schematic illustration of the phage-based cytosensor for cancer cells.

Before the fabrication of the cytosensor, continuous cyclic voltammetric (CV) scanning of the AuEs in H_2_SO_4_ solution were conducted to make the AuEs almost inclusion-free and activated. Subsequently, bio-recognition elements were immobilized on the biosensor surface by the layer-by-layer assembly of 3-mercaptopropionic acid (MPA), 1-ethyl-3-(3-dimethylaminopropyl) carbodiimide hydrochloride (EDC)/*N*-hydroxysuccinimide (NHS) and phages (Fig. 1). To monitor the fabrication process of biosensor and the recognition of analytes on the electrode surface, we adopted EIS method, which has been widely used for monitoring the changes of electrode surface state because of its sensitive response and rapid label-free detection^16^,^46^. In addition, as a nondestructive technique, EIS was also considered appropriate for phage-based sensor^13^. Due to its nontoxicity for living cell, [Fe(CN)_6_]^3−/4−^ solution was used as the redox probe^23^. To analyze the data of EIS, the Nyquist diagram and the Randles equivalent circuit model were applied^15^,^16^. The semicircle diameter of the Nyquist diagram corresponds to the electron transfer resistance (R_et_) deriving from insulativity of the electrode surface. Therefore, R_et_ indicates the change of the insulative layer which hinders the transfer of redox probe to the electrode surface.

**Figure 2:**
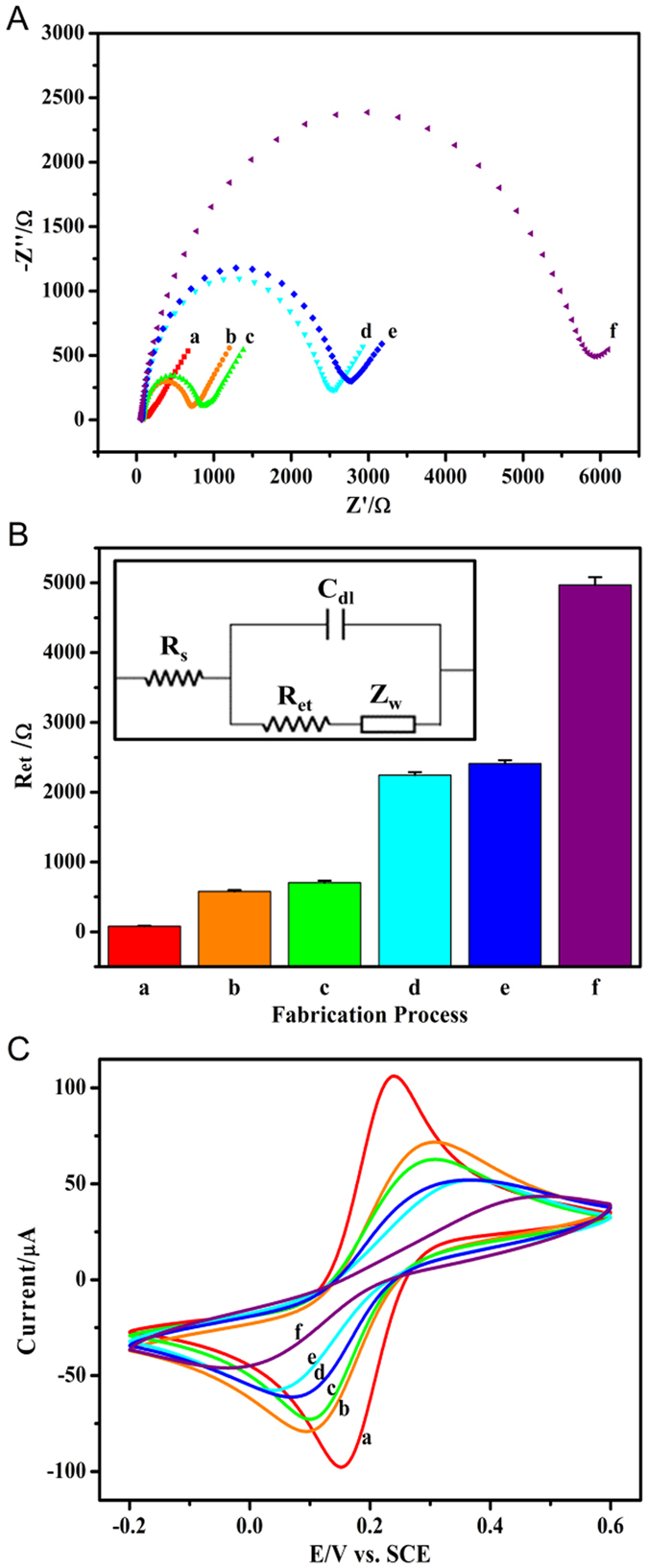
(**A**) Typical Nyqusit diagrams of EIS for fabrication and application of the cytosensor in [Fe(CN)_6_]^3−/4−^ solution. EIS results were recorded (a) at bare Au electrode pretreated electrochemically in 0.5 M H_2_SO_4_, (b) after formation of MPA layer, (c) after activation with EDC/NHS, (d) after immobilization of specific peptide-fused phages, (e) after blocking with BSA and (f) after immunological recognition with SW620 cells. Applied potential: +0.2 V (vs. SCE). Frequency range: 10^−1^  Hz–10^5^ Hz. (**B**) R_et_ for above steps (a–f) fitted to the Randles equivalent circuit model (Inset). C_dl_, double layer capacitor; R_et_, electron transfer resistance; Z _w_, Warburg resistor; R_s_, solution resistor. (**C**) Typical CV curves for above steps.

As shown in Fig. 2A, Nyquist diagrams changed incrementally with the successive modification of the Au electrode surface. Based on the Randles equivalent circuit model, the impedance data were fitted into corresponding electronic elements, and the successively changed R_et_ values were intuitively illustrated by histogram (Fig. 2B). After a series of pretreatments, the bare Au electrode surface was cleaned and activated, and exhibited a good electron transfer rate with the R_et_ value of 79.9 ± 6.4 Ω. Then MPA was covalently attached onto the Au electrode surface to form a carboxyl monolayer by the strong interaction of Au−S bonds, and the R_et_ value increased to 578 ± 21 Ω, indicating the formation of insulative layer of MPA. Whereafter, the carboxyl groups were immediately activated by EDC/NHS and the R_et_ value mildly increased to 703 ± 29 Ω. With the assembly of phage, the R_et_ value remarkably increased to 2245 ± 42 Ω, indicating the success of covalent immobilization of phage onto the surface of Au electrode by condensation reaction between carboxyl group and amine group. The remarkable increment in R_et_ could be due to sufficient binding time and the optimal concentration of phage, as well as the expected hindrance of the redox probe [Fe(CN)_6_]^3−/4−^ to the electrode surface. To avoid nonspecific physical adsorption and covalent binding between amino groups on cell surface and residual activated carboxyl of MPA, the sensor was blocked with 0.2% bovine serum albumin (BSA) in PBST. The nonionic surfactant 0.05% Tween-20 in PBST can allow to partly avoid unwished nonspecific adsorption^42^. The mild increase of R_et_ to 2411 ± 49 Ω suggested that most of activated carboxyl of MPA had covalently bound with phage. For immunoassay, the target SW620 cells were captured via a highly specific interaction between fusion octapeptides of phages and protein markers of cells. A high R_et_ of 4970 ± 114 Ω was observed, indicating that the large cells were immobilized onto the Au electrode surface to form a greater physical barrier between redox probe and the electrode surface.

Meanwhile, CV curves also provided the consistent information about the electrode surface (Fig. 2C). Using [Fe(CN)_6_]^3−/4−^ as the electrochemical redox probe, a pair of reversible redox peaks were observable for bare AuE (Fig. 2C, curve a), demonstrating a cleaned and activated electrode surface. Due to the coverage of MPA and blockage of active sites on AuE, the response current obviously reduced (Fig. 2C, curve b)^47^. With the further incubation of EDC/NHS mixture, the negative charged carboxyl group of MPA was bound with EDC and then replaced by NHS. Because the introduction of positive or neutral succinimide group of NHS further restrained the transfer of negative redox probe [Fe(CN)_6_]^3−/4−^ to the electrode surface, the peak current further reduced (Fig. 2C, curve c)^48^. Further, with the successive assembly of phages, BSA and cells, the CV responses gradually reduced until the current peaks were not obvious (Fig. 2C, curves d–f), indicating the coverage of insulative layers.

**Figure 3:**
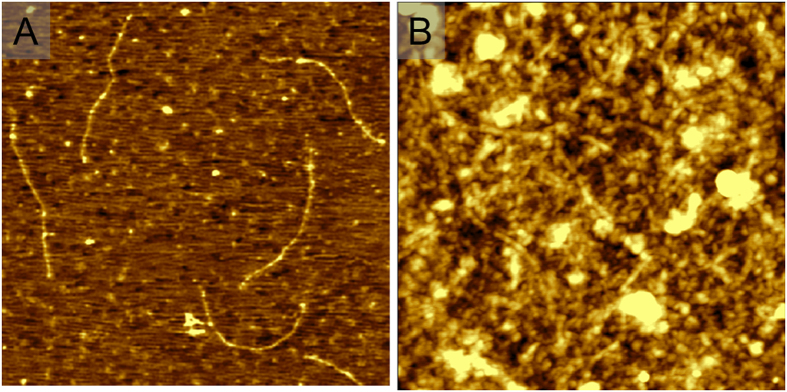
Typical AFM images acquired in air. (**A**) The unsaturated phage layer on gold surface (3 μm × 3 μm) after incubation in phage solution for 10 min. (**B**) The saturated functional phage layer on gold surface (2 μm × 2 μm) after incubation in phage solution for 6 h, and further blocking with 0.2% BSA.

To further study the topography of the functional surface of electrode after the above-mentioned assembly steps, the atomic force microscopic (AFM) measurements were conducted. Several filamentous phages with the length of ca. 1.3 μm and diameter of ca. 7 nm were observed on the gold surface for the case of the unsaturated phage incubation (Fig. 3A). The dense phage layer was clearly observed after incubation in saturated phage (Fig. 3B). The phages or phage bundles were covalently attached onto the gold surface to form an intercrossing random network. In addition, some spheroid particles on the layer could presumably be derived from coiling of filamentous phage^49^.

### Optimization of Conditions of Fabrication and Recognition Processes

**Figure 4:**
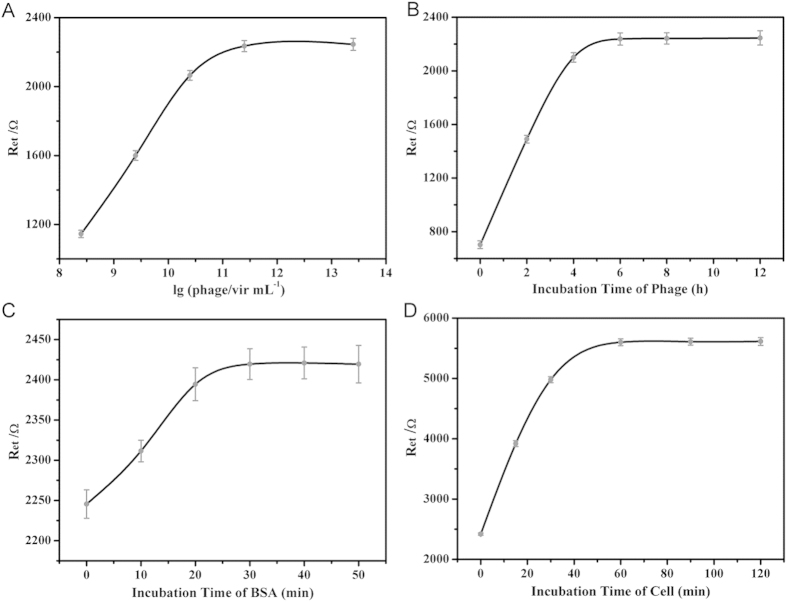
Effects of (**A**) phage concentration, (**B**) incubation time of phages, (**C**) incubation time of BSA, and (**D**) incubation time of SW620 cells on the EIS responses under different optimal conditions for fabrication of cytosensor.

To optimize cytosensor fabrication, we studied some important parameters, including phage concentration, incubation time of phage, incubation time of BSA, which were related to the cells capture capabilities and recognition efficiency of cytosensor. First, the modified electrodes were incubated in different phage concentrations (2.5 × 10^8^ − 2.5 × 10^13^ vir/mL) overnight, and the EIS spectra were recorded. EIS responses exhibited an upward tendency from 2.5 × 10^8^ to 2.5 × 10^11^ vir/mL, while no obvious change was observed for higher concentrations (Fig. 4A). Therefore, phage concentration of 2.5 × 10^11^ vir/mL was used for electrode modification. Subsequently, incubation time of phage (2.5 × 10^11^ vir/mL) was optimized. EIS responses increased with incubation time and reached a plateau at 6 h (Fig. 4B), indicating the amount of phages immobilized on electrode was saturated after 6 h. Because plentiful phages modified on the electrode would be eventually beneficial for cell recognition, 6 h was chosen as the optimal incubation time for phage immobilization. As for the blocking with BSA, the R_et_ showed a rising tendency with the increasing incubation time until 30 min (Fig. 4C). Hence, the blocking of sensor was accomplished after 30 min, which was selected as the optimal blocking time.

Since the incubation time of analytes could directly affect the electrochemical signal, we studied the effect of increasing incubation time of cells on EIS responses. As shown in Fig. 4D, R_et_ exhibited the continuous rise during 1 h incubation, indicating that more and more cells were captured by the cytosensor with the time accumulation. However, there was no apparent change of the R_et_ value after 1 h, suggesting that the amount of cells captured on the surface of the cytosensor was saturated. Hence, the optimal incubation time for the reaction between specific phage and antibody was 1 h.

### Quantitative Detection of Cells by Phage-Based EIS Cytosensor

**Figure 5:**
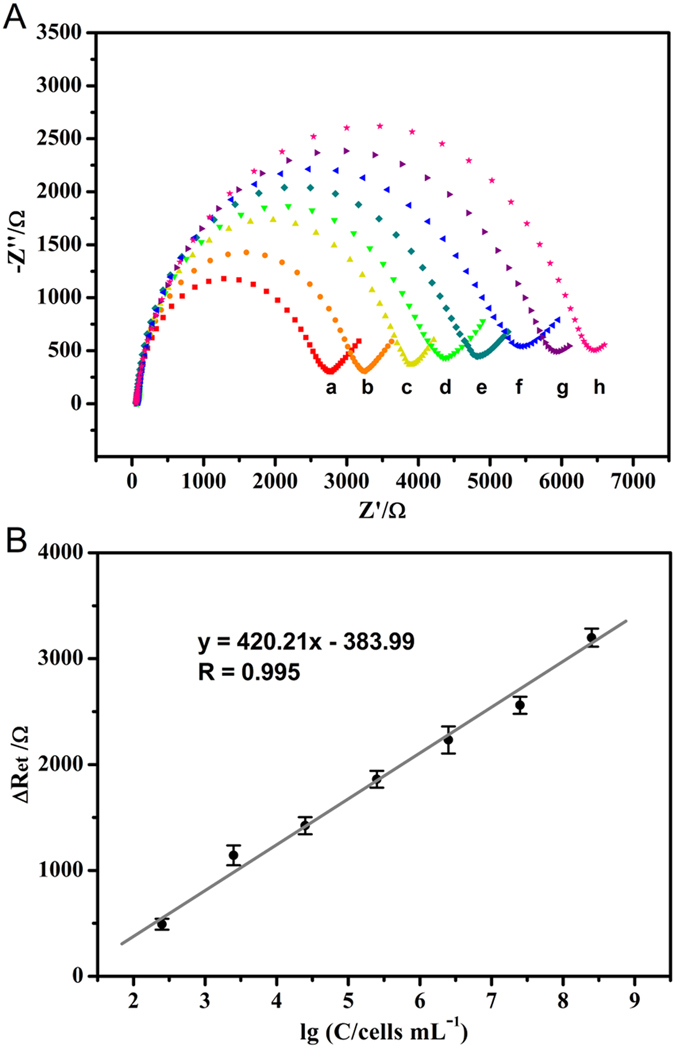
(**A**) EIS responses of fabricated cytosensors after immunological recognition with different concentrations of SW620 cells: (a) 0, (b) 2.0 × 10^2^, (c) 2.0 × 10^3^, (d) 2.0 × 10^4^, (e) 2.0 × 10^5^, (f) 2.0 × 10^6^, (g) 2.0 × 10^7^, and (h) 2.0 × 10^8^ cells mL^−1^. (**B**) The calibration curve for SW620 cells: the ΔR_et_ as a function of the logarithm values of SW620 cell concentration.

To investigate the performance of cytosensors for quantitative detection of tumor cells, we used EIS analytical system. EIS response directly reflected the electron transfer efficiency between the electroactive redox probe [Fe(CN)_6_]^3−/4−^ and the electrode surface, and the shielding effect of the cell layer to redox probe. In other words, the R_et_ values of the cytosensors relied on the amount of covalently immobilized cells and the recognition performance of phage. As shown in Fig. 5A, EIS responses changed gradually with the increasing cell concentration from 200 to 2.0 × 10^8^ cells mL^−1^, indicating the amount of the redox probes which penetrated to the electrode was gradually reduced. After fitting the EIS data into R_et_ values by the equivalent circuit model, it could be clearly observed that the R_et_ values sharply increased under low cell concentration, while slightly increased at the high cell concentration. The increment in R_et_ could be defined as ΔR_et_ = R_i_ − R_0_, where R_i_ is the R_et_ value after incubation of cell, and R_0_ is the R_et_ value after blocking with BSA but before cell detection. Through the further data processing, we found that ΔR_et_ values were proportional to the logarithm of the concentration of cell (Fig. 5B). The linear regression equation was ΔR_et_ (Ω) = 420.21 lg C (cells mL^−1^) − 383.99 with the correlation coefficient (R) of 0.995. The wide linear range of 2.0 × 10^2^ −2.0 × 10^8^ cells mL^−1^ (equivalent to 2.0 − 2.0 × 10^6^ in 10 μL assay volume) and the low limit of detection (LOD) of 79 cells mL^−1^ (S/N = 3) were obtained. The LOD was lower than those values of most cytosensors reported so far, such as 794 HeLa cells mL^−1^ at aptamer-based impedance sensor^50^, 100 HeLa cells mL^−1^ at folic acid-based electrochemical cytosensor^51^, 90 HL-60 cells mL^−1^ at small-molecule ligand-based impedance sensor^52^, 620 BGC-823 cells mL^−1^ at enzyme-linked tetrapeptide-based sensor^53^ and 1.8 × 10^4^ Jurkat cells mL^−1^ at poly-lysine/GCE impedance sensor^54^ (Table S1). These results demonstrated that the covalent immobilization of specific phage led to highly efficient capture of tumor cells onto the electrode surface. Therefore, the fabricated sensor was sensitive for detection of targeted cells.

### Evaluation of the Cytosensor Performance

**Figure 6:**
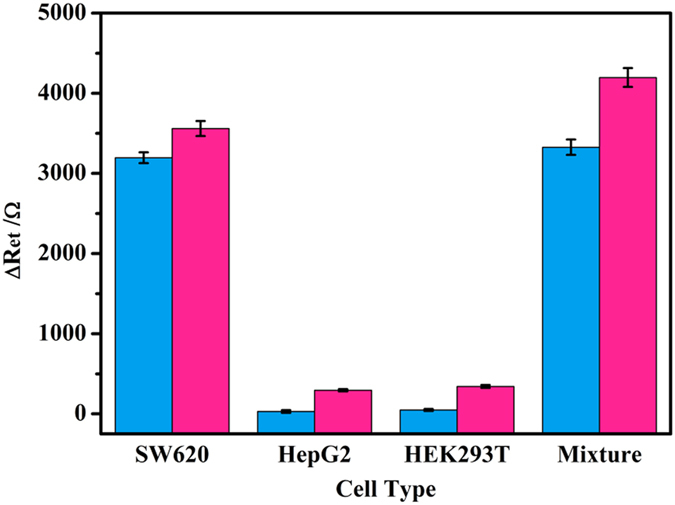
The typical ΔR_et_ of the proposed cytosensors (blue) and non-BSA-blocking cytosensors (red) after respective incubation with SW620 human colorectal carcinoma cells, HepG2 hepatoma carcinoma cells, HEK293T human embryonic kidney cells and the mixture of above-mentioned cells in serum. All cells were at the same concentration of 2.0 × 10^8^ cells mL^−1^.

To investigate the specificity of the proposed cytosensor for SW620 cell detection, control cells including HEK293T and HepG2 were regarded as potential interferents. Control cells and target cells SW620 were respectively incubated with the as-prepared cytosensor at the same condition. As for the as-prepared cytosensors, no obvious ΔR_et_ values were observed for interferents in comparison with the sharp ΔR_et_ value for SW620 (blue column in Fig. 6), demonstrating high specificity of the fabricated cytosensor and eliminable physical adhesion of cells onto electrode. Considering the complexity of the real blood, the ΔR_et_ value for the mixture of cancer cells (SW620 and HepG2), healthy cell (HEK293T) in human serum was detected, which showed the relative error of +4.1% in comparison with the case of SW620 only. Therefore, the proposed cytosensor had excellent reliability for real sample. Meanwhile, the non-blocking sensors were also applied in the same series of experiments to verify the effect of blocking with BSA (red column in Fig. 6). It could be observed that, compared with the BSA-blocked cytosensor, all the non-blocking sensors showed an obvious increase in ΔR_et_ value, indicating the blocking effect prevented nonspecific adsorption on electrode surface. These results confirmed not only the high selectivity of the phage-based cytosensor but also the necessity of blocking for sensor.

As the significant parameters of biosensor, the inter- and intra-assay reproducibility has to be investigated. On one hand, SW620 cells (2.0 × 10^5^ cells mL^−1^) were detected for five independent experiments by using different freshly prepared cytosensors. The relative standard deviation (RSD) was 4.9%, indicating acceptable reproducibility of the fabricated cytosensors. On the other hand, two different concentrations of SW620 cells were detected for five replicate detections. The RSD was 3.1% and 2.6% for 2.0 × 10^5^ and 2.0 × 10^7^ cells mL^−1^, respectively, indicating good precision of the cytosensor for the detection of cell.

In addition, the fabricated cytosensor remained 92.4% of its initial signal response after 30 days storage at 4 °C, suggesting the satisfactory storage stability, which is superior to antibody- or aptamer-based cytosensor. This excellent stability could be attributed to the fact that the landscape phage can be stored indefinitely at 4–8 °C^39^.

## Discussion

One of the major challenges in the design of biosensors for cancer diagnosis is to develop a low-cost and selective probe that can recognize overexpressed cancer cell receptors. Here, we combined the phage display technology and EIS technology to develop a label-free cytosensor for the detection of cancer cells for the first time, without complicated purification of recognition elements. Experimental results demonstrated that the phage-based EIS cytosensor for SW620 colorectal carcinoma cells was label-free, ultrasensitive, selective, reliable and stable, benefitting from the inherent merits of both phage-displayed specific octapeptides and EIS technology.

On one hand, it is noteworthy that the multivalency and structure of landscape phage as the display scaffold of specific probes made significant contributions. First, due to the avidity effect of multicopy display on the surface of the phage, homologous octapeptides could exhibit enhanced affinities and high specificity^55^. Second, the filamentous structure of fd phage is flexible to present appropriate conformation for the interaction with the large-size cancer cell. Meanwhile, one cell generally has many biomarkers for recognition ligand^52^. This means that more recognition sites at cell could combine with more specific octapeptides at the flexible filamentous phage, and the recognition process would be more efficient and the binding was firmer. Third, the specific octapeptides on the phage scaffold were highly stable in complex environments so as to make the corresponding sensor stable and repeatable^39^. Fourth, landscape phage is harmless to animal cells and could enhance the biocompatibility of sensor. On the other hand, the superiority of the fabricated sensor was also derived from the inherent merits of EIS technology. First, since EIS technique can monitor the minute impedance changes of the electrode interface, the fabricated impedance cytosensor was ultrasensitive and label-free. Second, EIS is a nondestructive technique due to the small amplitude perturbation, leading to a stable and reliable biosensor. These superiorities were not met for other recognition elements. With regard to the phage-based biosensor, Penner *et al*. developed the M13 phage-based impedance biosensor for detection of antigen^49^. However, it is not clear regarding the specificity of the M13 phage. Further, the phage probe-based impedance biosensors have not yet been applied in the detection of cells. It is highly desirable to develop novel biosensors for cancer cells. Additionally, our strategy to screen specific probes based on our f8/8 landscape phage display library was more effective, because there are 4000 copies of specific octapeptides on one phage surface. That is to say, there are 4000 recognition sites for the targets, which could not be met by other phage display methods. Therefore, it is possible to realize the sensitive and selective detection of target cells.

In addition, the fabrication procedure of the cytosensor was simple. First, large-scale preparation of specific peptide-fused phage was simple and without complicated purification procedures^39^. Second, phage display provided a filamentous phage “carrier” for the specific probe and reduced the complicated steps of polypeptide immobilization (which generally required synthesis and purification of the free peptide, attachment of a linker to the biosensor and finally conjugation of the peptide) to the single phage bioconjugation step^42^,^53^. Third, the EIS technology makes the practical application of the label-free sensor free from the complicated process including the binding of label with specific molecular and the recognition of specific molecular to target cell.

In conclusion, we have presented strategy for specific peptide-fused phage-cytosensor for ultrasensitive detection of cancer cells. The proposed cytosensor exhibited wide linear detection range, low limit of detection, high specificity, good reproducibility and satisfactory storage stability. The excellent analytical performance of the phage-based sensor could be attributed to good biocompatibility of recombinant phage, high specificity of surface displayed octapeptides, nanostructure of fibrous phages themselves and the inherent merits of EIS technology. The phage-based label-free impedance cytosensor with accessible phage recognition elements, simple operation, excellent analytical performance, would provide alternative technique for cancer cell detection.

## Methods

### Chemicals and Materials

MPA, 2-(N-morpholino)-ethanesulfonic acid (MES), EDC and NHS were purchased from Sigma-Aldrich (St. Louis, USA). BSA, Dulbecco’s Modified Eagle Medium (DMEM) and fetal bovine serum (FBS) were obtained from Solarbio (Beijing, China). Tween 20 and polyethylene glycol (PEG) were from Sinopharm Chemical Reagent Co., Ltd. (Shanghai, China). All the other reagents were of analytical grade and were used as received without further purification. All solutions were prepared with Milli-Q water (18.0 MΩ cm). [Fe(CN)_6_]^3−/4−^ solution was prepared with 10 mM K_3_Fe(CN)_6_, 10 mM K_4_Fe(CN)_6_ and 0.1 M KCl (as the supporting electrolyte) in sodium phosphate buffer (pH 7.4) and used as redox probe in the electrochemical detection system.

### Cell Culture

Cultures of the target cells (SW620) and control cells (HEK293T and HepG2) have been described previously^45^. Briefly, the cells were cultured in the flask containing DMEM supplemented with 100 μg/ml penicillin, 100 μg/ml streptomycin and 10% FBS. After a period of culture in a humidified cell incubator with 5% CO_2_ at 37 °C, cells were trypsinized with 0.25% trypsin in PBS, collected by centrifugation at 1000 rpm for 5 min, and then washed twice with PBS buffer. The cell pellets were suspended as stock and diluted to different concentrations with PBS buffer for electrochemical measurements. The final concentration of stock was measured by a Petroff-Hausser cell counter.

### Selection of the SW620 Cell-Specific Phages from the f8/8 Phage Library

The details for the selection of specific phage-displayed octapeptide by biopanning can be found in our previous work^45^. The preparation of the phage with the fusion octapeptides has been described previously by our co-workers^35^,^36^,^56^. The selected phages were simply amplified and cloned by inflecting *E. coli* K91 BlueKan host cells. Finally, phages were purified by PEG/NaCl precipitation, and then titrated^57^.

### Fabrication of the Electrochemical Cytosensor

Before modification of the AuEs (3 mm in diameter), the AuEs were polished with 0.02 μm alumina slurry on microcloth pad, and washed ultrasonically with water. The AuEs were cleaned and activated by CV in 0.5 M H_2_SO_4_ solution for 40 cycles within the potential range of 0 − + 1.5 V at a scan rate of 0.1 V/s. After washing of AuEs, the cytosensors were fabricated by layer-by-layer self-assembly process. First, the AuEs were incubated in 0.25 M MPA aqueous solution for 12 h to form a saturated MPA monolayer followed by washing with water^57^. The carboxyl group of MPA was then activated by incubating the modified electrode in 0.1 M MES buffer (pH 5.0) containing 50 mM EDC and 30 mM NHS for 5 h in order to convert the terminal carboxylic group of MPA to active NHS ester^57^. Next, the modified electrodes were rinsed with PBS and incubated in phage solution (2.5 × 10^11^ vir/mL) at 4 °C for 6 h to bind the amino group of phage surface with carboxyl group of MPA. After thorough washing with the PBST to remove any unbound phage, the modified electrodes were blocked with 0.2% BSA in PBST for 30 min to reduce nonspecific binding of analytes or impurities. For the detection of cells, 10 μL of cell suspensions were dropped onto the surfaces of the modified electrodes and incubated at 37 °C under a humid condition for 1 h. Before electrochemical measurement, the phage-based electrochemical cytosensors were gently and thoroughly washed with PBS to remove any non-specifically adsorbed cells.

### Apparatus and Measurements

UV absorption spectrum of displayed phage solution was performed on UV-1800 spectrophotometer (Shimadzu, Kyoto, Japan). Electrochemical measurements including EIS and CV were performed on a CHI 660D electrochemical workstation (CH Instruments, Chenhua, Shanghai, China) with a conventional three-electrode system composed of a modified electrode as a working electrode, a platinum wire as an auxiliary electrode and saturated calomel electrode (SCE) as reference electrode. All electrochemical measurements were carried out in [Fe(CN)_6_]^3−/4−^ solution. The EIS spectra were recorded at 0.2 V within the frequency range of 10^−1^−10^5^ Hz and the amplitude of 5.0 mV. AFM measurement was conducted in air at ambient pressure and humidity on the Agilent 5400 AFM system with tapping mode at scan rate of 0.5 line/s. Data analysis was performed with the Agilent technologies Picoview software 1.8.

## Additional Information

**How to cite this article**: Han, L. *et al*. A Label-Free Electrochemical Impedance Cytosensor Based on Specific Peptide-Fused Phage Selected from Landscape Phage Library. *Sci. Rep*. **6**, 22199; doi: 10.1038/srep22199 (2016).

## Supplementary Material

Supplementary Information
