# Supplementary material for: A Label-Free Electrochemical Impedance Cytosensor Based on Specific Peptide-Fused Phage Selected from Landscape Phage Library
Source: Sci Rep. 2016 Feb 24;6:22199. doi: 10.1038/srep22199 (PMC4764921; doi:10.1038/srep22199)
Supplement: Supplementary Information [file srep22199-s1.pdf]

**SUPPLEMENTARY INFORMATION**

# A Label-Free Electrochemical Impedance Cytosensor Based on Specific Peptide-Fused Phage Selected from Landscape Phage Library

Lei Han[1,2], Pei Liu[2], Valery A. Petrenko[3], Aihua Liu[1,2,*]

[1]Institute for Biosensing & In-Vitro Diagnostics, and College of Medicine, Qingdao University, 38 Dengzhou Road, Qingdao 266021, China

[2] Laboratory for Biosensing, Qingdao Institute of Bioenergy & Bioprocess Technology, Chinese Academy of Sciences, 189 Songling Road,Qingdao, 266101, China

[3] Department of Pathobiology, College of Veterinary Medicine, Auburn University, 269 Greene Hall, Auburn, Alabama 36849-5519, United States

*Correspondence and requests for materials should be addressed to A.H.L. (liuaihua@hotmail.com; liuah@qdu.edu.cn)

**Table of Contents**

**Figure S1.** The typical UV spectrum of purified phage. …………………….……...S2

**Table S1.** Comparison of Various Cytosensors for Detection of Human Cells .....S3

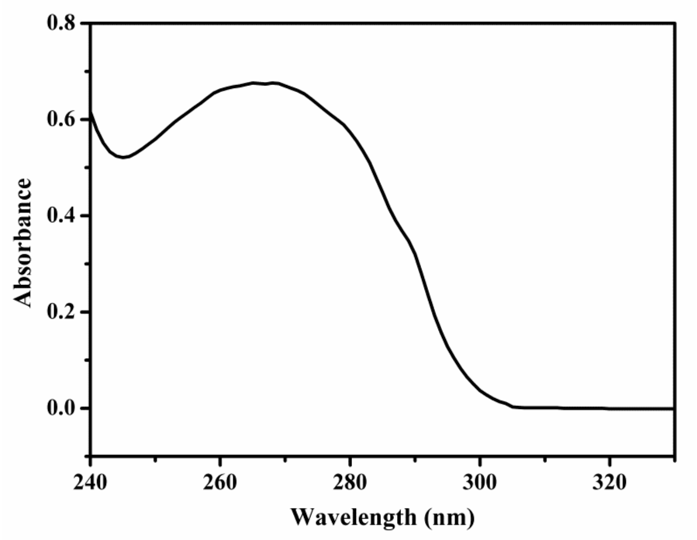


**Figure S1.** The typical UV spectrum of purified phage.

**Table S1.** Comparison of various cytosensors for detection of human cells

| Cytosensor | Cell | Linear range (cells $ml^{-1}$) | LOD (cells $ml^{-1}$) | Reference |
|---|---|---|---|---|
| Aptamer/PTCA/CRGO/GCE impedance sensor | Hela | $1.0 \times 10^3 - 1.0 \times 10^6$ | 794 | 1 |
| Enzyme-linked Con A/ 3D-architecture interface sensor | Hela | $8.0 \times 10^2 - 2.0 \times 10^7$ | 500 | 2 |
| FA/PAMAM/AuNPs/GCPE cytosensor | Hela | $1.0 \times 10^2 - 1.0 \times 10^6$ | 100 | 3 |
| VTA@MWCNTs impedance cytosensor | HL-60 | $2.7 \times 10^2 - 2.7 \times 10^7$ | 90 | 4 |
| FA/PEI/CMC-G impedance sensor | HL-60 | $5.0 \times 10^2 - 5.0 \times 10^6$ | 500 | 5 |
| SWCNTs-AuNPs-gelatin impedance sensor | HL-60 | $1.0 \times 10^4 - 1.0 \times 10^7$ | $5.0 \times 10^3$ | 6 |
| Enzyme-linked -peptide-SWNTs/GCE sensor | BGC-823 | $1.0 \times 10^3 - 1.0 \times 10^7$ | 620 | 7 |
| PLL/GCE impedance sensor | Jurkat | $5.0 \times 10^4 - 1.0 \times 10^7$ | $1.8 \times 10^4$ | 8 |
| Au-cage/$Ru(bpy)_3^{2+}$-Con A electrochemiluminescence sensor | K562 | $5.0 \times 10^2 - 5.0 \times 10^6$ | 500 | 9 |
| APBA-MWCNTs/GCE impedance sensor | K562 | $1.0 \times 10^3 - 1.0 \times 10^7$ | $1.0 \times 10^3$ | 10 |
| CNF/CS/GCE impedance sensor | K562 | $5.0 \times 10^3 - 1.0 \times 10^7$ | $1.0 \times 10^3$ | 11 |
| Aptamer-nanoparticle strip biosensor | Ramos | $4.0 \times 10^3 - 2.0 \times 10^5$ | 800 | 12 |
| Aptamer-CdSe NPs-PDDA photoelectrochemical cytosensor | CEM | $1.6 \times 10^2 - 1.6 \times 10^3$ | 80 | 13 |
| Phage-based impedance sensor | SW620 | $2.0 \times 10^2 - 2.0 \times 10^8$ | 79 | this work |

PTCA, 3,4,9,10-perylene tetracarboxylic acid;

CRGO, chemically reduced graphene oxide;

FA, folic acid;

PAMAM, poly(amidoamine);

GCPE, glassy carbon paste electrode;

VTA, 2-p-aminophenyl-1, 3, 2-dithiarsenolane;

MWCNTs, multi-walled carbon nanotubes;

PEI, polyethyleneimine;

CMC-G, carboxymethyl chitosan-functionalized graphene;

SWCNTs, single walled carbon nanotube;

AuNPs, gold nanoparticles;

PLL, poly-lysine;

APBA, 3-aminophenylboronic acid;

CNF, carbon nanofiber;

CS, chitosan;

PDDA, poly(dimethyldiallylammonium chloride).

## References:


1. Feng L, Chen Y, Ren J, Qu X. A graphene functionalized electrochemical aptasensor for selective label-free detection of cancer cells. *Biomaterials* **32**, 2930-2937 (2011).
2. Zhang J-J, Cheng F-F, Zheng T-T, Zhu J-J. Design and implementation of electrochemical cytosensor for evaluation of cell surface carbohydrate and glycoprotein. *Anal. Chem.* **82**, 3547-3555 (2010).
3. Tepeli Y, Demir B, Timur S, Anik U. An electrochemical cytosensor based on a PAMAM modified glassy carbon paste electrode. *RSC Advances* **5**, 53973-53978 (2015).
4. Xu Y, *et al.* Sensitive detection of tumor cells by a new cytosensor with 3D-MWCNTs array based on vicinal-dithiol-containing proteins (VDPs). *Biosens. Bioelectron.* **66**, 321-326 (2015).
5. Yang G, Cao J, Li L, Rana RK, Zhu J-J. Carboxymethyl chitosan-functionalized graphene for label-free electrochemical cytosensing. *Carbon* **51**, 124-133 (2013).
6. Zhang J-J, Gu M-M, Zheng T-T, Zhu J-J. Synthesis of Gelatin-Stabilized Gold Nanoparticles and Assembly of Carboxylic Single-Walled Carbon Nanotubes/Au Composites for Cytosensing and Drug Uptake. *Anal. Chem.* **81**, 6641-6648 (2009).
7. Cheng W, Ding L, Lei J, Ding S, Ju H. Effective cell capture with tetrapeptide-functionalized carbon nanotubes and dual signal amplification for cytosensing and evaluation of cell surface carbohydrate. *Anal. Chem.* **80**, 3867-3872 (2008).
8. Huang B, Jia N, Chen L, Tan L, Yao S. Electrochemical impedance spectroscopy study on polymerization of L-lysine on electrode surface and its application for immobilization and detection of suspension cells. *Anal. Chem.* **86**, 6940-6947 (2014).
9. Ge L, Su M, Gao C, Tao X, Ge S. Application of Au cage/$Ru(bpy)_3^{2+}$ nanostructures for the electrochemiluminescence detection of K562 cancer cells based on aptamer. *Sensors Actuators B: Chem.* **214**, 144-151 (2015).
10. Zhong X, Bai H-J, Xu J-J, Chen H-Y, Zhu Y-H. A Reusable Interface Constructed by 3-Aminophenylboronic Acid-Functionalized Multiwalled Carbon Nanotubes for Cell Capture, Release, and Cytosensing. *Adv. Funct. Mater.* **20**, 992-999 (2010).
11. Hao C, Ding L, Zhang X, Ju H. Biocompatible Conductive Architecture of Carbon Nanofiber-Doped Chitosan Prepared with Controllable Electrodeposition for Cytosensing. *Anal. Chem.* **79**, 4442-4447 (2007).
12. Liu G, Mao X, Phillips JA, Xu H, Tan W, Zeng L. Aptamer−Nanoparticle Strip Biosensor for Sensitive Detection of Cancer Cells. *Anal. Chem.* **81**, 10013-10018 (2009).
13. Zhang X, Li S, Jin X, Li X. Aptamer Based Photoelectrochemical Cytosensor with layer-by-layer assembly of CdSe semiconductor nanoparticles as photoelectrochemically active species. *Biosens. Bioelectron.* **26**, 3674-3678 (2011).
